# Published Research on Burnout in Nursing in Spain in the Last Decade: Bibliometric Analysis

**DOI:** 10.3390/healthcare8040478

**Published:** 2020-11-12

**Authors:** Ana Belén Barragán Martín, María del Mar Molero Jurado, María del Carmen Pérez-Fuentes, María del Mar Simón Márquez, Maria Sisto, José Jesús Gázquez Linares

**Affiliations:** 1 Department of Psychology, Faculty of Psychology, University of Almería, 04120 Almería, Spain; abm410@ual.es (A.B.B.M.); mmj130@ual.es (M.d.M.M.J.); mpf421@ual.es (M.d.C.P.-F.); msm112@ual.es (M.d.M.S.M.); ms168@ual.es (M.S.); 2 Department of Psychology, Faculty of Psychology, Universidad Politécnica y Artística del Paraguay, Asunción 1628, Paraguay; 3 Department of Psychology, Universidad Autónoma de Chile, Providencia 7500000, Chile

**Keywords:** burnout, nursing, bibliometrics, co-authorship network

## Abstract

Scientific production in the last decades has evidenced an increase in burnout syndrome in healthcare professionals. The objective of this bibliometric study was to analyze scientific productions on burnout in nurses in 2009–2019. A search was made on the Web of Science database on burnout in nursing. The variables evaluated were number of publications per year, productivity based on the journal and relationships between authors. Data were analyzed using Bibexcel software, and Pajek was used to visualize the co-authorship network map. A total of 1528 publications related to burnout in nurses were identified. The years with the most productivity were 2016 to 2017, when the publication rate increased noticeably over previous years. The Spanish journal with the most production on the subject was *Atención Primaria*. The co-authorship network analyzed illustrated collaboration patterns among the researchers. Scientific publications on the subject have increased in recent years due to problems in the healthcare system, which is in need of prevention and intervention programs for healthcare professionals.

## 1. Introduction

Hospitals are working daily to improve the quality of patient-centered medical attention [1,2,3] and factors that predict it [4], and employees providing such care perceive work as more emotional when they manage their own feelings and emotions [5]. Thus, nurses are not only in charge of attention and care of patients but are also a therapeutic tool in user care [6] and, therefore, carry a heavier load and are under greater pressure, and are even sometimes the subject of violence from users [7]. Emotional work has negative effects on members of medical attention organizations, such as lower job satisfaction [8,9], increased intention of rotation with coworkers [10], poor sleep quality due to the negative relationship between the components of emotional intelligence [11,12,13] and burnout [14].

In recent decades, scientific evidence has demonstrated increased burnout in different populations [15,16,17,18], especially in healthcare personnel [19], so it is a challenge to healthcare systems in view of the global phenomenon that has driven such research in many countries [20,21].

This syndrome refers to the response to stressful factors in the workplace, although it is how individuals cope with and manage these factors that causes burnout [22]. It is also defined by emotional fatigue and feelings of detachment from work and of inefficacy [23]. It is important to study the presence of this severe occupational risk in employees because of its consequences to the individual and their work [24,25,26]. Thus, in public health and the institutions themselves, it becomes necessary to understand the variables that influence its development, prevention and treatment, to be able to increase productivity and lower job absenteeism [27,28].

The prevalence figures for burnout in healthcare differ, as it depends on the criteria employed to evaluate the construct [29,30] and related variables [31]. For example, the study by Rusca and Setyowati [32] showed high prevalence of burnout syndrome in nurses in eastern Java (Indonesia). In South Korea, nurses in an intensive care unit also showed high burnout levels—in particular, the youngest with less work experience [33]. Prevalence scores of 66.6% of healthcare workers affected by burnout were found in Spain [34], while a study by Grau-Martin, Flichtentrei, Suner, Prats and Braga [35] found the prevalence in Spanish healthcare workers to be 14.9%. Álvarez, Mori and Gómez [36] also found a high percentage of nursing professionals (43.67%) with burnout. Several studies in the same country have also shown high levels of prevalence of burnout in nursing professionals [37,38]. Thus, the prevalence of burnout in Spanish nurses would be around 18% to 33%, showing the magnitude of the problem [39].

Burnout in nurses is linked to work overload, while self-efficacy and self-esteem act as protective variables [40]. Molero, Pérez-Fuentes, Gázquez, and Barragán [41] found that the level of nurses’ self-esteem differentiates them with respect to burnout and were able to identify different burnout profiles by self-esteem, empathy and social support. Empathy, job satisfaction and personality also influence burnout, which negatively intervene in the quality of care, derived from lack of self-confidence, lack of attention, low self-esteem [42,43,44] and job dissatisfaction [45].

The presence of this subject in the media has contributed to increasing knowledge, which can be measured through the various studies. Lately, qualitative and quantitative studies have been found in the literature on already published documents. These are metric studies that measure production by querying documents and analyzing such literature [46]. Such bibliometric analyses can evaluate the impact and number of publications in journals about a subject of study over time [47]. At the same time, co-authorship networks in the production are analyzed to find out their relationship in the most important studies in the field.

Bibliometric analyses have been done on many subjects in health [48,49,50], and some have undertaken burnout as the main theme [51,52]. However, up to now, burnout syndrome has been analyzed along with other constructs and in different professions, so the subject is receiving more international attention, and productivity on it in the literature is high. The most visible countries in scientific production are those considered most productive worldwide. The United States is in the lead [52], while scientific production on burnout in Spain has grown with daily increase in the syndrome’s presence in Spanish society. In the last decade, after the financial crisis of 2008, caused by the collapse of the real estate bubble in the United States in 2006, work conditions changed, and not only in that country—the repercussions were felt around the world, and also in Spain. Disproportional cuts were made in resources and in services, negatively affecting the quality and efficiency of healthcare and increasing the impact of burnout [53,54], especially in service professions such as nursing [55]. Those first government measures reduced material and human resources, lengthened the workday, decreased nursing personnel, lowered salaries, and so forth. This generated deterioration in attention to users at health centers and a work overload for employees. All these measures configured a new scenario, where the work environment caused demotivation and deterioration of healthcare professionals [56]. In 2019, the World Health Organization [57] called the burnout syndrome a work-related illness that worsened people’s physical and mental health. Therefore, scientific production has a fundamental role in the development of health policies [58,59], as these publications can form the basis for informing and contextualizing public health debates. At the same time, this type of bibliometric publication not only answers to the study of science and the evolution of scientific production, it also shows editorial management. The study of scientific production enables inquiry and comparison of changes that have been occurring in the burnout syndrome in the scope of nursing and the commitment and collaboration networks that have been established based on the parameters developed in each publication.

Studies in Spain have analyzed the burnout syndrome in the healthcare environment. However, it must be known what research on burnout in nurses in the country has been published and what variables related to this construct have appeared in articles in recent years before this public health problem can be approached. Thus, the objective of this study was to perform a metric analysis of the scientific production on burnout in nurses in Spain in 2009–2019.

This metric analysis also proposed the following specific objectives: (1) identify the journals where articles related to the subject are published, (2) determine the productivity of authors and their collaboration networks and (3) find out the study variables related to this syndrome.

## 2. Methods

To respond to our objectives, this study used the scientific method in which analysis provides indicators for analyzing the progress and current state of a certain subject matter. It was therefore carried out in five stages: recovery, migration, analysis, visual representation and interpretation.

In the first stage, *Recovery*, the sources and resources were selected; search and selection. The second stage, *Migration*, extracted, loaded, screened and processed the data. *Analysis* involves the scientific analysis and quantitative treatment of scientific and bibliometric indicators. *Visual Representation* of the parameters and identification follows. Finally, *Interpretation* is where the data are described, compared and contextualized.

First, a search was made on the Web of Science database for publications containing the words “burnout” and “nursing” in the title published anywhere in the world during 2009–2019, and then only studies done in Spain.

A series of filters were applied to the search based on the objective: as for the type of document, only articles were considered, and in a search period from 2009 to 2019. The type of source was limited to articles in English and Spanish published in journals. Furthermore, to find the publications only in Spain, it was also filtered by country. The results extracted were imported as unformatted text.

The following search equation was used for the Web of Science: TS = (burnout AND nursing), which found a total of 1528 articles. Filtering by country resulted in 123 studies carried out in Spain, all of them open access.

Based on the objectives posed, the inclusion search criteria set were: (1) English or Spanish language; (2) on burnout in nursing; (3) empirical studies only. For exclusion, criteria were: (1) not in English or Spanish; (2) public information articles, letters to editor, Ph.D. theses or documents not published in scientific journals; (3) duplicate studies; (4) related to subjects other than burnout syndrome in nursing.

Then, an in-depth review of these 123 studies was conducted to select only quantitative studies and analyze the type of research, instruments and related variables. The following inclusion criteria were set for this: only quantitative empirical studies that included the descriptors “burnout” and “nursing” in the title or in the abstract, thereby discarding 64 studies because they did not include both descriptors and 11 more because they were systematic reviews or meta-analyses. In the end, 48 studies were selected.

### 2.1. Procedure

Indicators for study variables selected were chronological production, production by document type, institutional production, editorial production, production by language and distribution according to the most commonly used scientific production laws.

### 2.2. Data Analysis

The analyses of the ISIWoS database were performed separately, as they were downloaded in different files to be able to read them with that program. Bibexcel software (HistCite Software LLC, New York, USA) [60] was used for this because of its flexibility and capability for managing a large volume of data and preprocessing them, and because the studies were extracted as text. The data found in Bibexcel were then processed by the Pajek program [61] to develop network maps. Given the large number of authors, the Pajek program was set to select only those with three or more co-authorships to form the co-authorship network. That is, authors who did not have at least three publications with another author were excluded.

The ATLAS.ti software (ver. 8.4, Scientific Software Development, Berlin, Germany) was used for analysis of the content, classifying input by the variables dealt with in the articles found in the first stage of the study.

## 3. Results

The results of the search showed that there were 1528 documents in the Web of Science database on scientific production related to burnout in nursing, and of these, after filtering, 123 documents pertained to research carried out in Spain. When the results had been extracted, their relevance was analyzed to see if the publications found were related to the main subject of our study, and three of them were eliminated from the search due to not meeting the inclusion criteria.

The results are presented below in three sections. Section 3.1 analyzes all the scientific production on burnout, the number of publications per year, the journals where these articles were published and the production level of journals and authors, using the 123 studies to do so. In Section 3.2, of the 123 documents, only the 48 which were quantitative studies are analyzed for type of research, instruments, and number of related variables. Finally, in Section 3.3, co-authorships and the variables related to burnout are analyzed (*n* = 123).

### 3.1. Number of Publications Per Year and Selection of Journals

**Figure 1:**
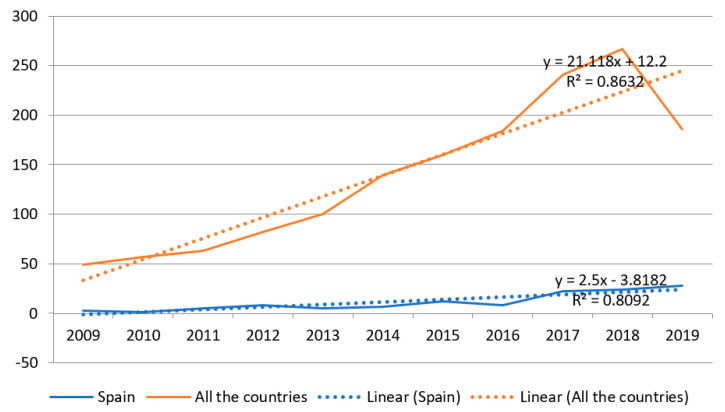
Scientific production on burnout in nursing based on the Web of Science (WOS) database and its trend during the years analyzed.

Figure 1 shows that international and national scientific production has been increasing over the years. In less than four years, the number of studies related to burnout in nursing has doubled. The first five years, 2009–2013, were less productive than the second five, in 2014–2018, for which the number publications was not representative (Table 1). Furthermore, the biggest boom in production was in 2016 to 2017, when 57 more studies were published internationally than in the previous year, and 13 more Spanish articles. Although in Spain, the number of articles on this subject was also found to increase over the years. Of the 123 documents found in Spain, 87 were published in English and 36 in Spanish. The trend in the number of publications in upcoming years will also increase as the R^2^ is 0.863 and 0.809.

Table 2 shows the Price Index by number of bibliographic references of articles published in 2009–2010 and the number of publications in Spain each year. With the exception of the last three years, the Price Index has remained rather low at 8.83% of total references.

**Figure 2:**
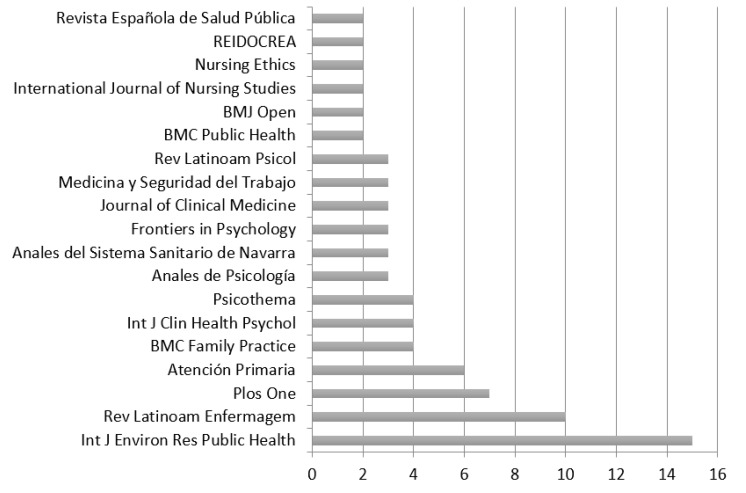
Journals that published the most Spanish studies of burnout in nursing.

In addition, a total of 62 journals published articles on burnout in nurses, 65.04% of them in the journals shown in Figure 2. The journal with the most publications in the period 2009–2019 was the *International Journal of Environmental Research and Public Health*. In Spain, the leading journal with the most production on the subject was *Atención Primaria.*

**Figure 3:**
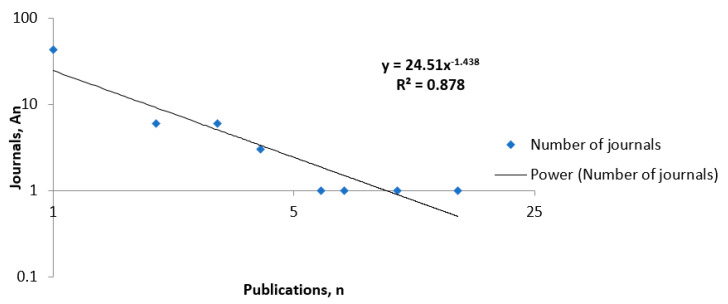
Application of Lotka’s law to productivity of journals on burnout in nursing.

The productivity data found in the journals on Spanish studies on burnout in nursing were compared applying Lotka’s law. During this period, a single journal had 15 publications on this subject, while 43 journals only had one. The R^2^ is 0.878, which is very close to 1, so the line is practically identical to the literature (Figure 3).

**Figure 4:**
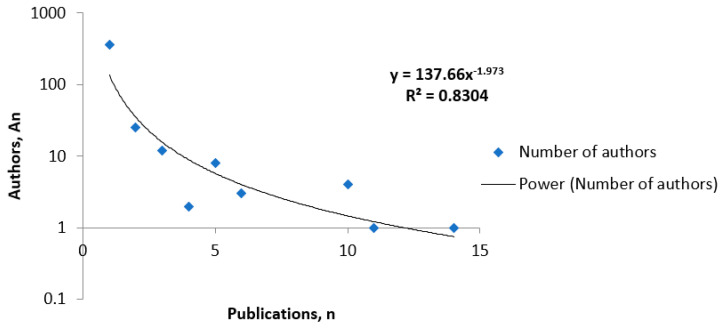
Distribution of Lotka’s law of productivity of authors.

Similarly, Figure 4 shows the result of applying Lotka’s law to the productivity of the authors, where the line is similar to the theoretical with an R^2^ near 1, at 0.830. Therefore, for both distributions, Lotka’s law shows that the number of authors or journals “*An*” who have published “*n*” studies on this subject is inversely proportional to *n* squared.

Table 3 shows author distribution by productivity, where most of them pertain to Productivity Level 1, which represents 86.67% of the total, and only 1.43% are on Productivity Level 3, with a total of six authors.

### 3.2. Review of Studies Performed in Spain

Of the 123 studies on the Spanish population, 48 were finally chosen for their analysis (see Table 4). Of these, 44 are cross-sectional or longitudinal studies on healthcare professionals, specifically nurses and/or certified nursing assistants. The majority of the studies were comprised of samples of nurses, and only four were conducted with a sample of nursing students. It should also be mentioned that 87.5% (*n* = 42) of the articles on burnout employed a cross-sectional design while only 4.2% (*n* = 2) were longitudinal.

The instruments used to evaluate burnout in these professionals in each of the studies were: 75% (*n* = 36) the various versions (MBI-GS, MBI-HSS and MBI-SS) of the Maslach Burnout Inventory (MBI); in 12.5%, the Brief Burnout Questionnaire (BBQ) and its multifactorial version CBB-M were used; the remaining 12.5% (*n* = 6) used questionnaires such as the Cuestionario Breve de Burnout (CBB) (Spanish Burnout Inventory), the Cuestionario de Burnout de Granada (GBQ) (Granada Burnout Questionnaire) or burnout questionnaires by the authors.

Table 4 shows that only 8.3% of experimental studies were controlled, randomized clinical trials which carried out an intervention with nurses and student nurses. One of them evaluated the effect of a mindfulness program and self-compassion on burnout and stress levels and the other evaluated the efficacy of intervention for the prevention and treatment of burnout in primary attention professionals.

Table 4 shows the number of variables used in the 48 studies, where the analysis found that 33% (*n =* 16) of the studies evaluated burnout along with another variable, which, in most cases, was empathy, followed by personality and job satisfaction, engagement, resilience and matters related to the conditions of effectiveness and communication at work. Overall, 20.8% (*n* = 10) of the studies related the syndrome with two of the variables above or, in their absence, added questionnaires and scales that measure health status, stress, anxiety and depression or intrapersonal variables, such as self-esteem, self-efficacy and emotional intelligence.

In the studies that employed four variables (6.3%), burnout, emotional intelligence, personality and perceived social support were related. Finally, in those that examined five or more variables, self-compassion, perceived stress, social and emotional loneliness were included, and scales other than the MBI were used to evaluate burnout.

### 3.3. Co-Authorship Map of Studies Done in Spain and Variables Related to Burnout

Co-authorship networks were found as a bibliometric indicator of collaboration to find out collaboration in each article—that is, how many authors wrote each article.

**Figure 5:**
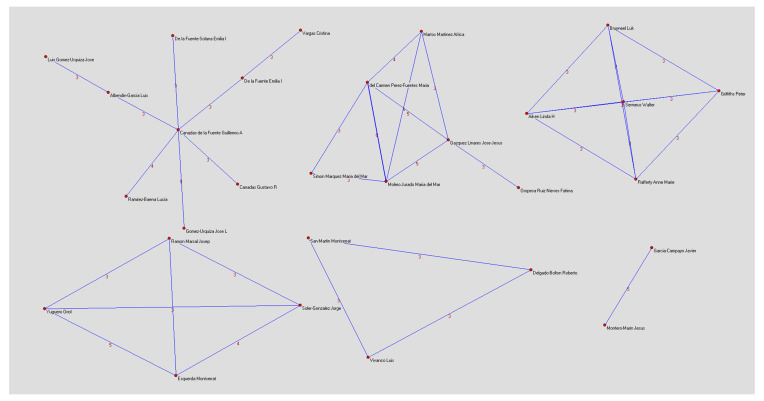
Co-authorship networks.

Figure 5 shows only six different co-authorship networks, as the Pajek program was set to show only three or more co-authorships. This figure shows that the co-authorship networks are different and not formed by the same number of authors. Therefore, each group was analyzed separately. The first co-authorship network was made up of researchers at the University of Granada who all belonged to the Information Processing and Decision-Making Group at the University of Granada in collaboration with the University of Valencia. The second network was made up of researchers in the University of Almería Psychology Department.

There is another mini-network made up of authors in Belgium and Pennsylvania, and finally, there are mini-networks made up of four or fewer authors belonging to institutions and universities in Catalonia and Madrid.

Most of the documents were written by more than one author, and so not only the national but also the international collaboration that configures these networks should be emphasized.

Finally, the content was analyzed for variables related to burnout in nursing and the number of studies found that contained those variables related to burnout. A total of 40 variables related to burnout were found, as shown in Table 5. The variables studied most in the studies analyzed were anxiety and depression (*n* = 4), emotional intelligence (*n* = 5), engagement (*n* = 5), empathy (*n* = 8), general health (*n* = 5), job satisfaction (*n* = 8), personality (*n* = 7), resilience (*n* = 4), self-efficacy (*n* = 4) and job stress (*n* = 3).

Table 5 shows that the majority of the variables related to burnout were not only factors associated with work. Many are individual or personal in scope, as these factors are determinant in its appearance. This is the case with anxiety or depression, personality, engagement, empathy, emotional intelligence, resilience, self-concept, self-efficacy, self-esteem, subjective social support and positive and negative effect. Depending on how they relate to burnout, they may act as protective or risk factors. On the other hand, the most important variables connected to the organizational context were job satisfaction, job tension and organizational behavior, and the variable which was fundamental in developing this syndrome was job stress. Most of the variables in the table are mainly related to emotional exhaustion.

## 4. Discussion

Burnout is one of the main problems found today in user attention and care professionals. Therefore, studies related to this syndrome in healthcare are of special interest. Burnout is increasingly present in scientific journals, especially starting in the last decade [54,55,56], and in particular since this syndrome has been considered an occupational disease [57]. Thus, this increase in production in recent years may respond to the prevalence of the syndrome in nursing, as shown by Álvarez et al. [36], who estimated that 43.67% of nurses have the syndrome. Although prevalence of the burnout syndrome is high in nurses in Spain [37,38], from around 18% to 33% [39], these percentages are similar to other countries [32,33].

This study showed uninterrupted growth in the number of publications, demonstrating the interest that the burnout syndrome generates in the national and international scientific community [58,59]. However, it should be kept in mind that although the figures found in 2019 on publications on this subject are no lower than in the year before, they may still be higher, as that was the year when this study was done and, therefore, the number of publications may have increased by year end. A bibliometric analysis on a subject enables major times or events related to the subject of study to be identified [46]. The percentages found by the Price Index are proportionate to the number of publications, since the boom in this subject began in 2016 to 2017 in Spain, so the highest values in references less than five years old were found in 2017 and later. However, this index is lower than for other studies in the field of occupational health [7], which may be because the search was limited to one database and, therefore, the range of references was reduced.

The *International Journal of Environmental Research and Public Health* was by far the most productive journal publishing articles on burnout in nursing [52]. This is because this journal publishes, annually, many more articles than the others. Furthermore, it is an interdisciplinary open-access journal, dealing with subjects on public health, environmental health, occupational hygiene, general health, etc., where the publication of manuscripts is faster than in other journals. In Spain, the journal with the highest production on the subject is *Atención Primaria*, which is devoted to primary health care.

The results also showed that studies done in Spain to-date are mostly cross-sectional observational studies where the prevalence is on knowing the condition of nurses with respect to the burnout syndrome, risk factors and protection from this symptom and its relationship with other variables [36,39], rather than studies conducted to prevent and intervene in symptoms or trials to reduce job stress and exhaustion. The instrument of excellence for measuring or evaluating burnout in these professionals was the Maslach Burnout Inventory (MBI) and two or three variables were employed in most cases, including the burnout syndrome, although it is true that a high percentage only evaluated burnout with descriptive analyses or validated a burnout questionnaire for healthcare professionals.

The results of the analysis of variables related to burnout found that empathy and job satisfaction had been studied most in recent years, as burnout is associated with therapeutic relations and care quality [42,43], followed by personality, in which low self-esteem and lack of self-confidence negatively affect job performance [44].

Part of the analysis was to find out the collaborations existing between authors in co-authorship networks in Spain [47]. In fact, a large number of networks were found but results were limited to show only collaborations between three or more authors in each article. Six of these were found. This is one of the limitations of this study, since it reduced the number of co-authorship networks, and therefore, data were excluded from the analysis that might have been relevant, as there are more collaborations by pairs of authors than by large groups. Another one of the main limitations of this study was the database search, as only publications in the Web of Science were included. Therefore, future research should widen the search to other databases to check whether the number of publications is the same or higher and also to see whether the authorship network is similar or. on the contrary, differs in the number of authors and collaborations. This study is a turning point for the preparation of future research which can take the scientific literature on the subject up to this time as a basis and see how it evolves. The results of bibliometric studies, such as this one, have also acquired considerable importance in elaborating scientific research policies and their management in the field of health.

The purpose of publications is so that the scientific community can compare, verify or reject the value of each study. Thus, knowing indicators in the areas of health sciences, such as co-authorship networks, journals and the research variables related to the burnout syndrome, gaps in the publications on the subject can be detected as well as what has to be designed and included in a new study with these indicators known as a reference. The findings of such a bibliometric study may be represented precisely and analyzed and will be of great use to society and the scientific community. It may be observed that, insofar as editorial management is concerned, scientific journals back studies related to the subject. Concerning research groups, it was observed that those specializing in this subject approached the analysis of burnout from different perspectives. Finally, knowing the variables related to this syndrome opens a range of possibilities in proposing new lines of research.

## 5. Conclusions

This study analyzed the literature on burnout in nurses, using the inclusive bibliometric search method. It also provided a first analysis of authorship collaboration networks in publications on burnout in nurses in Spain. Bibliometric studies provide a general view of the current state of a subject matter and the repercussion it has on society. Therefore, if we found that the number of publications on burnout in nurses has increased, it is because society and researchers are observing that there is a problem in the healthcare system which has to be analyzed and worked on for its prevention and intervention, as it is important to have an overview of the subject in order to be able to focus on valid strategies and open new lines of research.

Therefore, the results of this study have practical implications for both the individual and for healthcare organizations. There is still work to be done in identifying the factors that affect and/or relate to the burnout syndrome. This study shows the areas on which most studies have concentrated up to now, and based on this analysis, those where fewer studies have been done can be traced, and the direction can be provided for continued exploration after finding out other paths for creating tools that facilitate and reinforce the work of healthcare professionals. This would reduce the consequences derived from this syndrome and improve organizational and personal measures.

Finally, it may be said that bibliometric analysis can make a positive contribution to initiatives in the field of public health.

## Figures and Tables

**Table 1:** Number of publications per year.

Year	Number of Publications	Number of Publications in Spain
2009	49	3
2010	57	1
2011	63	5
2012	82	8
2013	100	5
2014	139	7
2015	160	12
2016	184	8
2017	241	22
2018	267	24
2019	186	28
Total	1528	123

**Table 2:** Price Index (% of references published less than five years ago).

Year	Number of Publications in Spain	Number of References	N References <5 Years	Price Index (%)
2009	3	75	(2005–2009) 1	1.33
2010	1	1	(2006–2010) 0	0
2011	5	209	(2007–2011) 3	1.43
2012	8	193	(2008–2012) 3	1.55
2013	5	183	(2009–2013) 6	3.27
2014	7	122	(2010–2014) 3	2.45
2015	12	244	(2011–2015) 9	3.68
2016	8	59	(2012–2016) 2	3.38
2017	22	259	(2013–2017) 33	12.74
2018	24	135	(2014–2018) 21	15.55
2019	28	38	(2015–2019) 38	100
Total	123	1518	119	7.83

**Table 3:** Distribution of authors by productivity level.

Productivity Level	N Studies	Authors	
		*n*	%
3	> or =10	6	1.43
2	2 to 9	50	11.9
1	1	364	86.67
Total		420	100

**Table 4:** Type of studies and instruments with samples of nurses.

Study Design											
**Observational**								**Experimental**			
Cross-sectional				Longitudinal				Clinical trials			
*n*		%		*n*		%		*n*		%	
42		87.5		2		4.2		4		8.3	
**Instruments**											
MBI				CBB				Other questionnaires			
*n*		%		*n*		%		*n*		%	
36		75		6		12.5		6		12.5	
**Number of variables per study**											
Analyses with one variable		Analyses with two variables		Analyses with three variables		Analyses with four variables		Analyses with five variables		Analyses with seven variables	
*n*	%	*n*	%	*n*	%	*n*	%	*n*	%	*n*	%
14	29.2	16	33.3	10	20.8	3	6.3	4	8.3	1	2.1

**Table 5:** Analysis by subject related to burnout in the publications.

Variables	Number of Studies	Variables	Number of Studies
Aggressive behavior	1	Organizational behaviors	1
Altruism	1	Perceived social support	2
Anxiety or depression Disorders	4	Personality profiles	7
Autonomy	1	Positive and negative effect	1
Care assessment	1	Practice environment of the nurses	1
Communication skills	2	Psychological disturbance	1
Competence	1	Relatedness	1
Compliance	1	Resilience	4
Coping	2	Satisfaction	3
Emotional intelligence	5	Self-compassion	2
Empathy	8	Self-concept	1
Empowerment	1	Self-conduct	1
Engagement	5	Self-efficacy	4
General health	5	Self-esteem	1
Health problems	1	Self-perceived health	1
Job satisfaction	8	Sleep quality	1
Job strain	1	Subjective social support	1
Loneliness	1	Trauma screening	1
Mental health	1	Wellness in the academic context	1
Mindfulness	1	Work stress	3
